# Visual outcomes and spectacle independence of a non-diffractive wavefront-shaping intraocular lens in post-LASIK patients

**DOI:** 10.3389/fmed.2025.1509889

**Published:** 2025-05-21

**Authors:** Wei Fan, Meiyi Zhu, Guangbin Zhang

**Affiliations:** ^1^School of Medicine, Xiamen Eye Center and Eye Institute of Xiamen University, Xiamen, China; ^2^Xiamen Clinical Research Center for Eye Diseases, Xiamen, Fujian, China; ^3^Xiamen Key Laboratory of Ophthalmology, Xiamen, Fujian, China; ^4^Fujian Key Laboratory of Corneal and Ocular Surface Diseases, Xiamen, Fujian, China; ^5^Xiamen Key Laboratory of Corneal and Ocular Surface Diseases, Xiamen, Fujian, China; ^6^Translational Medicine Institute of Xiamen Eye Center of Xiamen University, Xiamen, Fujian, China

**Keywords:** cataract, LASIK surgery, wavefront-shaping, depth of field, spectacle independence, EROF IOL

## Abstract

**Purpose:**

To compare visual outcomes, depth of field (DOF), spectacle independence, and patient satisfaction of cataract patients with and without previous myopic laser in situ keratomileusis (LASIK) surgery who received a non-diffractive extended range-of-focus (EROF) intraocular lens (IOL).

**Setting:**

Xiamen Eye Center, Xiamen, China.

**Design:**

Prospective case series.

**Methods:**

A total of 50 eyes of 41 patients implanted with the Vivity IOL were divided into Post-LASIK and Virgin groups. Outcome measures included uncorrected distance visual acuity (UDVA), corrected distance visual acuity (CDVA), uncorrected intermediate visual acuity (UIVA), uncorrected near visual acuity (UNVA), refractive outcomes, defocus curves, subjective DOF, spectacle independence, and Visual Function questionnaire (VF-14) after 3 months postoperatively.

**Results:**

Postoperatively, 70% of the Post-LASIK and 86.7% of the Virgin group had refractive error within ± 0.50 D (*P* = 0.28). The majority of both groups achieved 20/25 or better UDVA, with no significant differences between groups for UDVA, CDVA and UIVA (*P* > 0.05). The UNVA was significantly better in the Post-LASIK group (0.31 ± 0.08 logMAR) than Virgin group (0.45 ± 0.10 logMAR, *P* < 0.001). The Post-LASIK group showed a smoother curve with a wider landing area, and better subjective DOF compared to the Virgin group (*P* < 0.001). Spectacle independence at near ranges in bright light was higher in the Post-LASIK group (81.3 % vs 48 %, *P* = 0.033). Both groups reported high visual satisfaction, but the Post-LASIK group had fewer difficulties with near-distance tasks in the VF-14 questionnaire.

**Conclusion:**

In post-LASIK eyes, this wavefront-shaping EROF IOL was well-tolerated and provided an extended range of vision with significantly better UNVA, fewer difficulties in daily activities and higher spectacle independence for near vision compared to normal eyes.

## Introduction

Laser in situ keratomileusis (LASIK), one of the most widely performed corneal refractive surgeries worldwide, has a history spanning nearly 25 years. In the United States, over 20–25 million eyes have undergone LASIK surgery in the past 20 years. (1) However, patients who had LASIK in its early years are now reaching the age where cataracts commonly develop. Ophthalmologists are increasingly faced with the challenge of managing patients who have undergone myopic LASIK and now require cataract surgery. These patients often wish to pursue spectacle independence again and have higher expectations regarding the refractive outcome, due to their positive experiences with initial corneal refractive surgery. However, selecting a multifocal intraocular lens (IOL) for patients with reshaped corneas remains challenging. Although a history of LASIK surgery is not a contraindication for the use of multifocal IOLs, these patients typically have higher amounts of corneal higher-order aberrations (HOAs), spherical aberration (SA) and lower keratometry, which complicate IOL power prediction and can lead to refractive errors and inferior outcomes for those expecting spectacle independence (2–7). Additionally, many studies have shown that diffractive multifocal IOLs can decrease contrast sensitivity and induce adverse visual symptoms, such as glare and halos (8–10). Therefore, a non-diffractive EROF IOL is a reliable choice for post-LASIK eyes, as it has shown high tolerability to residual refractive errors and low photic phenomena because of its unique optical design (11, 12).

The AcrySof IQ Vivity IOL is a new non-diffractive wavefront-shaping EROF lens, designed with a patented X-Wave technology. According to the manufacturer, this EROF IOL consists of a 2.20 mm wavefront-shaping optic in the central part of the anterior surface to stretch and shift the wavefront without splitting light (12, 13). This design extends the focal range instead of creating multiple focal points, and appears to be less prone to image degradation and artifacts compared to diffractive IOLs while maintaining a functional range of vision (14, 15).

Previous studies of healthy eyes have confirmed that the Vivity IOL provides a continuous range of focus rather than discrete foci at specific distances, offering good visual acuity (VA) results for far and intermediate distances, though near vision was poorer compared to previous multifocal IOLs (16–20). Some in vitro experiments have also shown that the Vivity IOL exhibits minimal spurious light comparable to monofocal IOLs and features an estimated extended range of focus of 1.75 diopters (D) (21, 22). Therefore, this study aims to compare visual outcomes, subjective depth of field (DOF), spectacle independence, and patient satisfaction in patients with and without previous myopic LASIK surgery who received a non-diffractive wavefront-shaping EROF IOL.

## Materials and methods

### Study design

In this prospective clinical trial, 50 eyes from 41 patients underwent cataract surgery with the implantation of an EROF lens (AcrySof IQ Vivity) between September 2023 and March 2024 at the Department of Cataract, Xiamen Eye Center, Affiliate Xiamen University, China. They were divided non-randomly into two groups: 20 eyes of 16 patients with prior myopic LASIK surgery formed the study group (Post-LASIK), and 30 eyes of 25 patients without LASIK surgery formed the control group (Virgin).

All investigations adhered to the tenets of the Declaration of Helsinki, and informed consent was obtained from all patients. Approval was obtained from the ethics committee at the institution (Approval Number: XMYKZX-KY-2024-047). Patients were informed of the advantages of this non-diffractive EROF IOL and the potential problems, including the need for spectacle correction for certain activities, loss of contrast, and the requirement for sufficient light for adequate visual function.

### Inclusion and exclusion criteria

Inclusion criteria for the study group were as follows: a history of myopic LASIK surgery with a centered optical zone, visually significant cataracts interfering with daily activities, and implantation of a non-diffractive EROF IOL (AcrySof IQ Vivity). For the Virgin group, inclusion criteria included clinically significant age-related cataracts affecting daily functioning, no other ocular pathology, and no history of prior ocular surgery, with all patients receiving implantation of a Vivity IOL. Exclusion criteria were preoperative astigmatism exceeding 1.0 D in corneal topography, previous LASIK with small optical zones (5.0 mm or less), preoperative total irregular astigmatism, mainly corneal HOAs in the 4.0 mm zone of corneal topography higher than 0.6 D, and ocular pathologies that could potentially influence the postoperative refraction results.

### Preoperative and postoperative assessments

All patients underwent a routine preoperative ophthalmologic examination, including measurement of corrected distance visual acuity (CDVA) and uncorrected distance visual acuity (UDVA) at 4 m using the Early Treatment Diabetic Retinopathy Study charts under photopic conditions, uncorrected intermediate visual acuity (UIVA) at 66 cm, uncorrected near visual acuity (UNVA) at 40 cm, keratometry, axial length (AL), IOL power and target spherical equivalent (SE) by optical biometry (IOLMaster 700, Carl Zeiss Meditech AG), slit lamp evaluation and fundoscopy. Corneal tomography (Pentacam HR, Oculus Optikgeräte GmbH) was performed to confirm the regularity of the previous ablation and astigmatism, and to measure corneal HOAs (4.0 mm zone), spherical aberration (SA) in the 6.0 mm zone and pupil size.

Comprehensive, postoperative refractive measurements were performed at least 3 months after cataract surgery. At the last postoperative visit, the following parameters were measured: CDVA, UDVA, UIVA, UNVA, manifest refraction spherical equivalent (MRSE), mean prediction error (MPE), mean absolute error (MAE), and the percentage of eyes within ± 0.5 D, ± 1.0 D, ± 1.5 D, and ± 2.0 D of target refraction. The defocus curve was measured from +1.50 to −4.00 D in steps of 0.50 D. The MPE was defined as the postoperative SE minus the predicted residual refractive error, with positive values indicating a hyperopic shift and negative values indicating a myopic shift. VA was expressed in logarithmic minimum angle (logMAR). To minimize accommodative effects, patients were instructed to fixate at the designated testing distances with full fogging. All measurements were performed by the same experienced optometrist to ensure consistency.

### Subjective DOF assessments

Depth of field was defined as the range of focusing errors for which the image of the target appears to have the same clarity, contrast, and form as the optimal in-focus image (23, 24). Defocus curves could be used to measure subjective defocus tolerance for EDOF IOLs (25, 26). According to the peer-reviewed literature, criterions to define what is optimal or not, vary from 0.10 logMAR to 0.20 logMAR or 0.30 logMAR in pseudophakic eyes (26, 27). In our study, the subjective DOF was obtained from the defocus curve by identifying the range of vergences that provided a visual acuity value of ≤ 0.1 and 0.2 logMAR.

### Patient satisfaction and spectacle independence

To subjectively measure patient satisfaction, a translated, modified and validated Chinese version of the Visual Function Index (VF-14) questionnaire (see Supplementary materials) was used on a scale of 0–4 points (28). The Chinese-translated VF-14 matches the Chinese socio-cultural norms to enhance item comprehension. It includes items such as visual lifestyle activities (reading small print/newspaper/large font, recognizing familiar people, seeing stairs, reading signs, doing fine handwork, signing names, playing games, taking part in sports, cooking, watching TV, driving at day, and driving at night); and overall satisfaction (“Would you choose this IOL again?”). Responses for visual lifestyle activity items were scored on a five-point Likert scale from 0 (“No difficulty”) to 4 (“Unable to do the activity”). The response category “not applicable” was considered missing data, and the overall satisfaction with the IOL was either yes or no. Additionally, at the 3 months follow-up, spectacle independence was expressed using the Intraocular Lens Satisfaction (IOLSAT, ITT number: 60043935) questionnaire. This proprietary Alcon questionnaire asks subjects about their visual performance at various distances in both bright and dim light. All tests and evaluations were performed by the same group of professionals.

### Surgical technique

Cataract surgeries were performed by one experienced surgeon (G.B. Zhang) under topical anesthesia. A standardized phacoemulsification was performed through a 2.2 mm temporal corneal incision using the Centurion active-fluidics System (Alcon Laboratories, Inc.). The same EROF IOL (DFT015, AcrySof IQ Vivity) was inserted into the capsular bag. The first available negative-power IOL was selected using the Barrett true-K formula for post-LASIK eyes and the Barrett Universal-II formula for normal eyes, based on the optical biometry with the optimized constant provided by manufacturer. After surgery, all patients received the same treatment consisting of a combination of levofloxacin (Cravit) and dexamethasone (Tobradex) eye drops four times a day during the first week, and then gradually tapered over the following 3 weeks.

### Sample size

The sample size calculation was based on a previous study of cataract patients with prior myopic LASIK surgery who were implanted with an EDOF IOL (29). We selected UNVA as the primary outcome measure because achieving functional near vision without corrective lenses is not only a key performance indicator of non-diffractive EROF IOLs but also an important goal for many post-LASIK cataract patients, particularly given their history of seeking spectacle independence. In that study, the UNVA in the two groups were 0.13 ± 0.13 logMAR and 0.46 ± 0.10 logMAR, respectively. To detect a clinically significant difference between the two groups in our study, we used PASS 15.0.5 software to calculate the sample size based on the available data. The results showed that at least eight samples were required in each group, with a total of at least 16 samples needed for this study (alpha = 0.05 and power = 0.9).

### Statistical analysis

Descriptive values were given as the mean ± standard deviation. Data were tested for normal distribution using the Shapiro-Wilk test. An independent *t*-test was used to compare normally distributed variables between the groups. Non-normally distributed data were analyzed using the Mann–Whitney U test. Categorical data were compared using the Pearson chi-squared test. A *P*-value less than 0.05 was considered statistically significant. All statistical analyses were performed using SPSS statistical software (version 19.0, IBM SPSS, Inc.).

## Results

### Preoperative data

Overall, 41 patients (50 eyes) completed the study, with 16 patients (20 eyes) in the study group (Post-LASIK) and 25 patients (30 eyes) in the control group (Virgin). There were no significant differences between the two groups in terms of age (Post-LASIK: 52.4 ± 6.3 years, range 41–61; Virgin: 55.6 ± 11.1 years, range 27–73; *P* = 0.203), gender, CDVA, UDVA, UIVA, UNVA, IOL power, pupil size and target SE (all *P* > 0.05, Table 1). However, because of the history of prior LASIK surgery in the study group, significant differences were observed between the two groups in terms of mean keratometry, AL, corneal HOAs and SA (both *P* < 0.05). The preoperative data were summarized in Table 1.

**TABLE 1:** Patient demographics and preoperative data.

Parameter	Group		*P-*value
	Post-LASIK	Virgin	
Eyes	20	30	–
Mean age (y)	52.4 ± 6.3	55.6 ± 11.1	0.203
**Sex**			
Male	10	11	0.248
Female	6	14	–
Mean CDVA (logMAR)	0.49 ± 0.15	0.58 ± 0.23	0.191
Mean UDVA (logMAR)	0.72 ± 0.18	0.76 ± 0.20	0.471
Mean UIVA (logMAR)	0.52 ± 0.18	0.56 ± 0.17	0.461
Mean UNVA (logMAR)	0.63 ± 0.17	0.62 ± 0.21	0.880
Mean keratometry	39.68 ± 1.74	43.94 ± 1.35	< 0.001
Mean AL	26.93 ± 1.82	24.29 ± 1.38	< 0.001
HOAs	0.34 ± 0.12	0.25 ± 0.09	0.028
SA	0.70 ± 0.16	0.36 ± 0.16	< 0.001
Pupil size	3.01 ± 0.49	2.97 ± 0.46	0.736
IOL power	19.0 ± 2.9	18.8 ± 3.9	0.945
Target SE	−0.11 ± 0.07	−0.15 ± 0.09	0.135

CDVA, corrected distance visual acuity; UDVA, uncorrected distance visual acuity; UIVA, uncorrected intermediate visual acuity; UNVA, uncorrected near visual acuity; logMAR, logarithmic minimum angle; AL, axial length; HOAs, higher-order aberrations; SA, spherical aberration; IOL, intraocular lens; SE, spherical equivalent.

### Postoperative refraction

**FIGURE 1:**
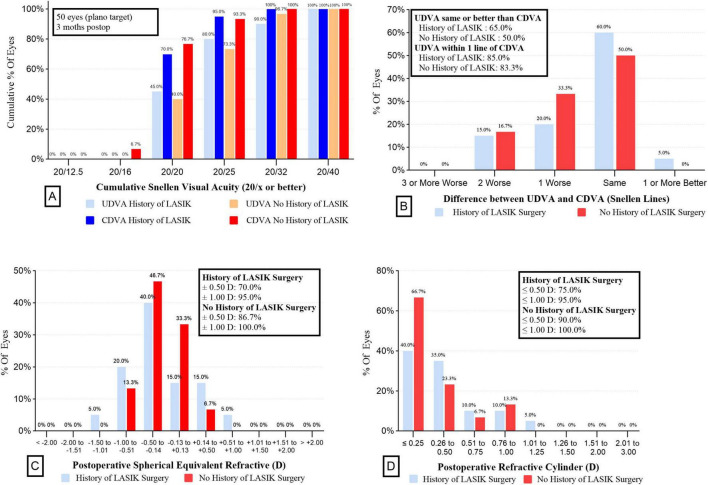
Refractive outcomes for wavefront-shaping EDOF IOL in the two groups. **(A)** UDVA and CDVA in the two groups. **(B)** Difference between UDVA and CDVA in the 2 groups. **(C)** Postoperative spherical equivalent refractive in the 2 groups. **(D)** Postoperative refractive cylinder in the 2 groups. EDOF, extended depth-of-focus; IOL, intraocular lens; UDVA, uncorrected distance visual acuity; CDVA, corrected distance visual acuity.

Figure 1 illustrates the visual and refractive outcomes. The majority of both groups achieved 20/25 or better UDVA (73.3% vs 80.0% for without a history of LASIK surgery vs with, *P* = 0.84) (Figure 1A). There were no significant differences in the UDVA, CDVA and UIVA between the two groups (all *P* > 0.05) (Table 2). Interestingly, the UNVA was significantly better in the Post-LASIK group (0.31 ± 0.08 logMAR) than in the Virgin group (0.45 ± 0.10 logMAR, *P* < 0.001). Postoperative MRSE showed that both groups achieved a slight myopic result as intended preoperatively, with no significant differences between the groups (*P* > 0.05, Table 2). There was a slight myopic shift in both groups regarding the MPE: −0.16 ± 0.46 D (range −1.07 to +0.68 D) for the Post-LASIK group and −0.09 ± 0.22 D (range −0.61 to +0.31 D) for the Virgin group. Because the MPE does not describe the performance as precisely as the MAE, only descriptive data without *P*-values were delivered (30). Furthermore, the MAE was higher in the Post-LASIK group (0.40 ± 0.27 D), with a statistically significant difference compared to the Virgin group (0.20 ± 0.14 D, *P* = 0.007) (Table 2). Postoperative refractive error was with in ± 0.50 D of plano in 70% of eyes with previous LASIK surgery and 86.7 % of eyes without LASIK surgery (*P* = 0.28) (Figure 1C). Likewise, the percentage of eyes with postoperative refractive cylinder of 0.50 D or less was 75% in the Post-LASIK group and 90% in the Virgin group (Figure 1D).

**TABLE 2:** Postoperative visual acuity and prediction error.

Parameter	Group		*P-*value
	Post-LASIK	Virgin	
UDVA (logMAR)	0.09 ± 0.10	0.09 ± 0.09	0.720
CDVA (logMAR)	0.04 ± 0.06	0.02 ± 0.07	0.283
UINA (logMAR)	0.18 ± 0.06	0.22 ± 0.07	0.110
UNVA (logMAR)	0.31 ± 0.08	0.45 ± 0.10	< 0.001
MRSE (D)	−0.28 ± 0.49	−0.24 ± 0.27	0.759
MPE (D)	−0.16 ± 0.46	−0.09 ± 0.22	–
MAE (D)	0.40 ± 0.27	0.20 ± 0.14	0.007

UDVA, uncorrected distance visual acuity; CDVA, corrected distance visual acuity; UIVA, uncorrected intermediate visual acuity; UNVA, uncorrected near visual acuity; MRSE, manifest refraction spherical equivalent; MPE, mean prediction error; MAE, mean absolute error; logMAR, logarithmic minimum angle; D, diopters.

### Defocus curves and DOF

**FIGURE 2:**
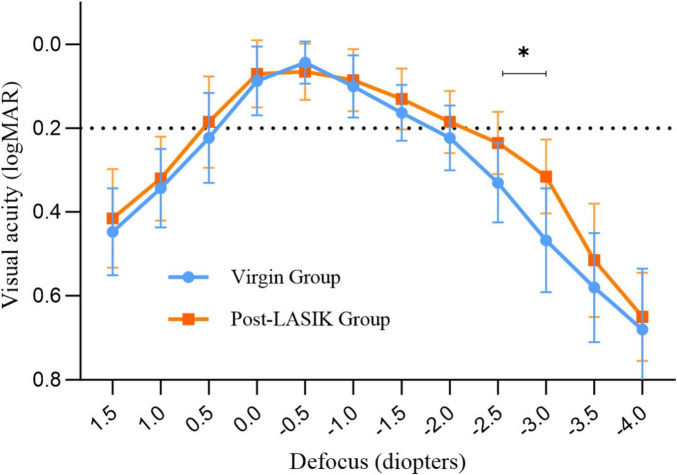
Monocular defocus curves for the two groups. *Indicates a statistically significant difference between the two groups at −2.5D and −3.0D.

Figure 2 shows the mean defocus curves of the two groups 3 months postoperatively. Both groups exhibited similar defocus curves, with maximum visual acuity close to 0 logMAR. Interestingly, the Post-LASIK group showed a smoother curve with a wider landing area compared to the Virgin group (Figure 2). The defocus VA from +1.5 to −2.0 D was not statistically significantly different between the two groups. However, at defocus curves of −2.5 and −3.0 D, the Post-LASIK group demonstrated significantly better VA than the Virgin group (both *P* < 0.01).

Mean DOF results for each group are represented in Table 3. Regardless of whether a 0.1 logMAR or 0.2 logMAR criteria was used to measure subjective DOF, the Post-LASIK group exhibited a better depth of focus than the Virgin group (all *P* < 0.05) (Table 3).

**TABLE 3:** Subjective DOF in two groups.

Group	Subjective DOF	
	0.1 logMAR criterion	0.2 logMAR criterion
Virgin	1.70 ± 0.66	2.67 ± 0.53
Post-LASIK	2.08 ± 0.82	3.38 ± 0.62
*P*-value	0.043	0.001

DOF, depth of field; logMAR, logarithmic minimum angle.

### Spectacle independence and patient satisfaction

Table 4 shows the postoperative levels of spectacle dependence in the two groups. There was no statistically significant difference in reported frequency of glasses use between the two groups for distance and intermediate vision in both bright light and dim light conditions (all *P* > 0.05). However, 81.3% (13 of 16 patients) in the Post-LASIK group reported higher levels of spectacle independence at near ranges, particularly in bright light, compared to the Virgin group [48.0 % (12 of 25 patients), *P* = 0.033] (Table 4). In dim light at near ranges, although the Post-LASIK group appeared to have a higher proportion of spectacle independence compared to the Virgin group (68.8% VS 48.0%, *P* = 0.192), this difference was not statistically significant. Overall, 75 % (17 of 24 patients) of the Post-LASIK group and 52 % (17 of 24 patients) of the Virgin group reported complete spectacle independence.

**TABLE 4:** Summary of Intraocular Lens Satisfaction (IOLSAT) questionnaire results.

Condition	Percentage of subjects never or rarely needing glasses (%)		*P*-value
	Post-LASIK (*N* = 16)	Virgin (*N* = 25)	
**Bright light**			
Distance (“far away”)	87.5	92.0	1.0
Intermediate (“arm’s length”)	93.8	88.0	0.948
Near (“up close”)	81.3	48.0	0.033
**Dim light**			
Distance (“far away”)	81.3	88.0	0.886
Intermediate (“arm’s length”)	75.0	84.0	0.760
Near (“up close”)	68.8	48.0	0.192
Overall	75.0	52.0	0.141

The VF-14 questionnaire was answered by all patients, as shown in Table 5. The questionnaire indicated a high level of satisfaction with daily life activities at far and intermediate distances for both groups (all *P* > 0.05) (Table 5). At near distances, patients in the Virgin group reported greater difficulty for reading small print, newspapers or fill out forms (mean scores: 2.64 ± 0.91, 1.92 ± 0.86, 1.20 ± 0.64, respectively) than the Post-LASIK group (1.81 ± 0.91, 0.63 ± 0.62, 0.63 ± 0.50, all *P* < 0.01). Finally, visual satisfaction was high for both groups, with 87.5% of post-LASIK patients expressing that they would undergo surgery again with the same type of IOL compared to 80% of the Virgin group (*P* = 0.844).

**TABLE 5:** Subjective scores of the VF-14 questionnaire 3 months postoperative.

Situation	Score (mean ± SD)		*P*-value
	Post-LASIK (*N* = 16)	Virgin (*N* = 25)	
**Far distance**			
1. Reading signs, such as traffic signs, street signs, store signs, advertising board, or plate number;	0.19 ± 0.40	0.12 ± 0.33	0.556
2. Taking part in sports, such as playing Ping-Pong or badminton, doing exercise, shadowboxing;	0.31 ± 0.48	0.28 ± 0.46	0.826
3. Watching TV;	0.25 ± 0.45	0.32 ± 0.48	0.635
4. Day driving such as automobile, motorcycle;	0.63 ± 0.50	0.60 ± 0.50	0.874
5. Night driving such as automobile, motorcycle;	1.13 ± 0.72	0.92 ± 0.76	0.388
**Intermediate distance**			
6. Reading large font, such as a large-print book newspaper, numbers on a telephone, wall clock;	0.38 ± 0.50	0.60 ± 0.64	0.288
7. Recognizing familiar people when they are close to you;	0.25 ± 0.45	0.36 ± 0.49	0.466
8. Seeing steps, stairs, or curbs;	0.44 ± 0.51	0.60 ± 0.71	0.567
9. Playing games, such as card games, mahjong, chess;	0.44 ± 0.51	0.56 ± 0.58	0.542
10. Cooking;	0.50 ± 0.52	0.56 ± 0.58	0.807
**Near distance**			
11. Reading small print, such as labels on medicine bottles, a telephone book, price list, watch;	1.81 ± 0.91	2.64 ± 0.91	0.007
12. Reading a newspaper or a book;	0.63 ± 0.62	1.92 ± 0.86	0.000
13. Signing your name or filling out forms.	0.63 ± 0.50	1.20 ± 0.64	0.005
Patient satisfaction			
14. Would you choose this IOL again?	87.5%	80.0%	0.844

VF-14, Visual Function Index -14; Score from 0 (“No difficulty”) to 4 (“Unable to do the activity”) for all items. The response category “not applicable” was considered missing data.

## Discussion

Nowadays, with the increasing availability of various IOL types and implantation strategies, selecting the appropriate IOL has become a complex task, particularly for special patient groups, such as those with a history of LASIK surgery. There is a widespread belief that implanting diffractive multifocal IOLs in post-LASIK patients carries risks due to increased corneal HOAs, inaccuracies in IOL power calculations, and reduced contrast sensitivity (31–33). Consequently, non-diffractive EROF IOLs appear to be a preferable choice. Firstly, these IOLs create one continuous elongated focus rather than several foci, making them more tolerant of postoperative residual refractive errors in post-LASIK patients. Additionally, the wavefront-shaping Vivity IOL, which incorporates negative SA, can counteract the positive corneal SA induced by myopic LASIK (6). To our knowledge, this is the first prospective and comparative study to report on the visual outcomes, subjective DOF, spectacle independence and patient satisfaction following the implantation of the non-diffractive EROF (Vivity) IOL in patients with and without previous myopic LASIK surgery.

As we know, corneal refractive surgery results in increased corneal HOAs and SA, and similar findings were observed in our study. Although there was a significant difference in HOAs between the two groups, we excluded cases with HOAs greater than 0.6 D (in the 4.0 mm zone) and those with off-center ablation to ensure more regular corneas and enhance the comparability of the data between the two groups. Residual refractive error is a common source of postoperative dissatisfaction following the implantation of advanced technology IOLs (34, 35). Christopher et al. reported that patients who had previously undergone refractive surgery and were implanted with EDOF IOLs achieved excellent outcomes, with 77% of eyes within ± 0.50 D (36). Similar results were observed by Palomino-Bautista et al. (37) with 61.6% of eyes being within ± 0.5 D of target refraction after LASIK surgery and subsequent EDOF IOL implantation. In this trial, 70% of eyes in the Post-LASIK group were within ± 0.5 D, which is slightly inferior to the Virgin group (86.7 %), but consistent with previous studies. Furthermore, the MAE was higher in the Post-LASIK group (0.40 ± 0.27 D) compared to the Virgin group (0.20 ± 0.14 D), indicating that IOL power calculation in patients who have undergone LASIK remains less predictable than in those with healthy eyes. Notably, there was no significant difference between the two groups in the number of eyes within ± 0.50 and ± 1.00 D of postoperative refractive error and refractive cylinder, suggesting that the non-diffractive EROF IOL provides good tolerance for postoperative refractive outcomes in post-LASIK patients.

Interestingly, our data showed that postoperative UNVA was better in the Post-LASIK group compared to the Virgin group, with significant differences in the defocus curves between the two groups at near distances (−2.5 D and −3.0 D). The defocus curve of the Post-LASIK group maintained a VA close to 0.3 logMAR (20/40 Snellen, 0.5 decimal), even at −3.0 D, and exhibited a smoother curve with a wider landing area than the Virgin group. Previous studies have demonstrated that patients with smaller pupils implanted with the Vivity IOL might benefit from the pinhole effect, which can enhance the wavefront-stretching effect (38, 39). In our study, there was no significant difference in pupil size between the two groups. Hence, we speculate that the reasons for better UNVA and a wider defocus curve in the Post-LASIK group may be twofold: firstly, our exclusion criteria limited the impact of high HOAs on visual quality; secondly, Cheng et al. (40) showed that SA, coma, and secondary astigmatism could expand the depth of focus. In our study, for this non-diffractive wavefront-shaping EROF lens, the increased corneal SA, changes in the anterior and posterior corneal surfaces, and flattening of corneal curvature following myopic laser surgery all contribute to an extended depth of focus.

Depth of field is one of the most crucial outcomes in our trial, as it indicates how well the IOL performs across patients with varying ocular conditions. According to the criteria of American Academy of Ophthalmology (AAO), an EDOF IOL should provide a monocular negative depth of focus of at least 0.5 D greater than that of a monofocal control at a 0.2 logMAR level. Some trials have shown that the non-diffractive wavefront-shaping technology meets the AAO EDOF criteria while limiting the level of visual disturbances (16, 41, 42). In our study, subjective DOF was defined as the range of distances on the defocus curve where VA remains above a predetermined value, such as 0.1 or 0.2 logMAR criterion. All subjective DOF measurements in both groups exceeding 1D of defocus imply the effectiveness of the EROF IOL properties. In a previous optical bench simulation of post-LASIK eyes, the Vivity IOL achieved a DOF of 2.54 ± 0.31 D, demonstrating considerable immunity to the presence of HOAs and maintaining a quite constant DOF for a large range of corneal positive SA (11). In our real-world clinical study, the subjective DOF in post-LASIK eyes, measured using the 0.1 logMAR and 0.2 logMAR crierion, was 2.08 ± 0.82 D and 3.38 ± 0.62 D, respectively, both significantly higher than in Virgin eyes. These results suggest that the Vivity IOL exhibits a larger range of DOF and appears particularly suitable for post-LASIK surgery eyes. Although limited information is provided by manufacturers about the optical function of this wavefront shaping IOL, some in vitro experiments have demonstrated that the EDOF IOL functions by increased spherical aberration of different order (22, 43, 44). Specifically, after corneal myopic LASIK surgery, changes in corneal asphericity and regularity, combined with the complex anterior surface design of the Vivity IOL, may contribute to the extended DOF considerably.

Consistent with previous studies (45, 46), our research demonstrated that both groups exhibited a high level of spectacle independence for far and intermediate distances, never or rarely needing glasses. However, at near distances in bright light, patients with prior LASIK surgery showed higher spectacle independence compared to Virgin group (81.3% vs 48%). This might be due to a better UNVA, a greater DOF or a lower expectation for improved outcomes after surgery in the Post-LASIK group. During preoperative discussions, the ophthalmologist likely emphasized the potential drawbacks of the IOL and the uncertain outcomes for these post-LASIK cataract patients. Consistently, the overall percentage of subjects reporting they rarely or never needed glasses was higher in patients with prior LASIK surgery (75% vs 52% in the Virgin group), driven primarily by higher percentages at near distances in both bright light and dim light conditions. In our study, although the MAE was significantly higher in post-LASIK patients, postoperative VF-14 scores, especially for near activities such as reading small print, reading newspapers, and signing names, were better than in Virgin group. Additionally, postoperative satisfaction with the non-diffractive EROF IOL was very high and, when interviewed, about 87.5% of patients said that they would choose the same IOL again. These findings also confirm the greater tolerance and broader indications of the Vivity IOL in patients who have undergone LASIK surgery.

A limitation of this study was that the patients included in the Post-LASIK group all had well-centered, regular corneal ablation patterns and were satisfied with the visual quality after laser surgery. These may have partially enhanced the effect of the Vivity IOL. Therefore, the authors emphasize that, the results of this study should not be generalized to patients with poor visual quality after refractive surgery, decentered ablations, or excessively high HOAs. Additionally, contrast sensitivity and photic phenomena were not measured in our study. Many studies have shown that visual disturbances with the Vivity IOL are similar to those with monofocal IOLs and superior to diffractive multifocal IOLs (14, 19, 20, 46). This study did not include these data as it was not focused on comparing different types of IOLs. Furthermore, another shortcoming of this non-randomized study was the small sample at a single center, along with an imbalance between the two groups, which may have introduced potential bias. To confirm our findings, a long-term prospective study with more participants from diverse groups of surgeons, hospitals, and races would be required to determine the actual differences between the two groups. Finally, the preoperative axial length differences between the two groups somewhat weaken the reliability of the study’s results. Nonetheless, this is a meaningful comparative study to confirm the safety and efficacy of the Vivity IOL implantation and to measure subjective DOF in patients with previous LASIK surgery.

With the introduction of this new class of non-diffractive EROF IOL, a highly satisfactory solution is provided for the individual patients, but it also emphasizes the need for meticulous patient selection. Further research is necessary to refine our understanding of how changes in HOAs and corneal SA after LASIK surgery may result in better near vision with EROF IOL.

## Conclusion

The Vivity IOL may be a viable option for patients with previous LASIK surgery who wish to reduce their dependence on glasses but are not candidates for multifocal IOLs. In cataract patients with prior LASIK surgery, this non-diffractive wavefront-shaping EROF IOL provided an extended range of vision with significantly better near vision while delivering similar distance and intermediate vision, a wider DOF, fewer difficulties for daily activities, and a higher rate of spectacle independence for near vision compared to normal eyes.

## Data Availability

The raw data supporting the conclusions of this article will be made available by the authors, without undue reservation.
