# Supplementary material for: Visual outcomes and spectacle independence of a non-diffractive wavefront-shaping intraocular lens in post-LASIK patients
Source: Front Med (Lausanne). 2025 May 21;12:1509889. doi: 10.3389/fmed.2025.1509889 (PMC12133751; doi:10.3389/fmed.2025.1509889)
Supplement: Supplementary file 1 [file Table_1.doc]

**Appendix 1.**

**The Chinese-translated version of the Visual Function Index (VF-14)**

**Questionnaire given after multifocal intraocular lens implantation**

**VF-1.**

Do you have any difficulty, even with glasses, reading small print, such as labels on medicine bottles, a telephone book, price list, watch?

YES ( ) NO ( ) Not applicable ( )

If yes, how much difficulty do you currently have?

A little ( ) A moderate amount ( ) A great deal ( ) Unable to do the activity ( )

**VF-2.**

Do you have any difficulty, even with glasses, reading reading a newspaper or a book?

YES ( ) NO ( ) Not applicable ( )

If yes, how much difficulty do you currently have?

A little ( ) A moderate amount ( ) A great deal ( ) Unable to do the activity ( )

**VF-3.**

Do you have any difficulty, even with glasses, reading large font, such as a large-print book or newspaper, numbers on a telephone or mobile phone, wall clock?

YES ( ) NO ( ) Not applicable ( )

If yes, how much difficulty do you currently have?

A little ( ) A moderate amount ( ) A great deal ( ) Unable to do the activity ( )

**VF-4.**

Do you have any difficulty, even with glasses, recognizing familiar people when they are close to you?

YES ( ) NO ( ) Not applicable ( )

If yes, how much difficulty do you currently have?

A little ( ) A moderate amount ( ) A great deal ( ) Unable to do the activity ( )

**VF-5.**

Do you have any difficulty, even with glasses, seeing steps, stairs, or curbs?

YES ( ) NO ( ) Not applicable ( )

If yes, how much difficulty do you currently have?

A little ( ) A moderate amount ( ) A great deal ( ) Unable to do the activity ( )

**VF-6.**

Do you have any difficulty, even with glasses, reading signs, such as traffic signs, street signs, store signs, advertising board, or plate number?

YES ( ) NO ( ) Not applicable ( )

If yes, how much difficulty do you currently have?

A little ( ) A moderate amount ( ) A great deal ( ) Unable to do the activity ( )

**VF-7.**

Do you have any difficulty, even with glasses, signing your name or filling out forms?

YES ( ) NO ( ) Not applicable ( )

If yes, how much difficulty do you currently have?

A little ( ) A moderate amount ( ) A great deal ( ) Unable to do the activity ( )

**VF-8.**

Do you have any difficulty, even with glasses, playing games, such as card games, mahjong, chess?

YES ( ) NO ( ) Not applicable ( )

If yes, how much difficulty do you currently have?

A little ( ) A moderate amount ( ) A great deal ( ) Unable to do the activity ( )

**VF-9.**

Do you have any difficulty, even with glasses, taking part in sports, such as playing Ping-Pong or badminton, strolling, doing exercise, shadowboxing?

YES ( ) NO ( ) Not applicable ( )

If yes, how much difficulty do you currently have?

A little ( ) A moderate amount ( ) A great deal ( ) Unable to do the activity ( )

**VF-10.**

Do you have any difficulty, even with glasses, cooking?

YES ( ) NO ( ) Not applicable ( )

If yes, how much difficulty do you currently have?

A little ( ) A moderate amount ( ) A great deal ( ) Unable to do the activity ( )

**VF-11.**

Do you have any difficulty, even with glasses, watching television?

YES ( ) NO ( ) Not applicable ( )

If yes, how much difficulty do you currently have?

A little ( ) A moderate amount ( ) A great deal ( ) Unable to do the activity ( )

**VF-12.**

Do you have any difficulty, even with glasses, day driving such as automobile, motorcycle, or nonmotorized vehicle?

YES ( ) NO ( ) Not applicable ( )

If yes, how much difficulty do you currently have?

A little ( ) A moderate amount ( ) A great deal ( ) Unable to do the activity ( )

**VF-13.**

Do you have any difficulty, even with glasses, night driving such as automobile, motorcycle, or nonmotorized vehicle?

YES ( ) NO ( ) Not applicable ( )

If yes, how much difficulty do you currently have?

A little ( ) A moderate amount ( ) A great deal ( ) Unable to do the activity ( )

**VF-14.**

Would you recommend this IOL to someone else?

Strongly not recommend ( ) Not recommend ( ) Recommend ( ) Strongly recommend ( )
